# Exercise during preoperative therapy increases tumor vascularity in pancreatic tumor patients

**DOI:** 10.1038/s41598-019-49582-3

**Published:** 2019-09-27

**Authors:** Claudia Alvarez Florez Bedoya, Ana Carolina Ferreira Cardoso, Nathan Parker, An Ngo-Huang, Maria Q. Petzel, Michael P. Kim, David Fogelman, Salvador Gabriel Romero, Huamin Wang, Minjeong Park, Matthew H. G. Katz, Keri L. Schadler

**Affiliations:** 1 Department of Pediatrics, The University of Texas MD Anderson Cancer Center, Houston, TX USA; 2 Department of Surgical Oncology, The University of Texas MD Anderson Cancer Center, Houston, TX USA; 3 Department of Behavioral Science, The University of Texas MD Anderson Cancer Center, Houston, TX USA; 4 Department of Palliative, Rehabilitation and Integrative Medicine, The University of Texas MD Anderson Cancer Center, Houston, TX USA; 5 Department of Gastrointestinal Medical Oncology, The University of Texas MD Anderson Cancer Center, Houston, TX USA; 6 Department of Anatomical Pathology, The University of Texas MD Anderson Cancer Center, Houston, TX USA; 7 Department of Biostatistics, The University of Texas MD Anderson Cancer Center, Houston, TX USA; 8 Departmento de Radiologia e Oncologia, Faculdade de Medicina, Centro de Investigacao Translacional em Oncolgia, Instituto do Cancer do Estado de Sao Paulo, Universidade de Sao Paulo, Sao Paulo, Brazil

**Keywords:** Pancreatic cancer, Tumour angiogenesis

## Abstract

The efficacy of chemotherapy is reduced by dysfunctional tumor vasculature, which may limit chemotherapy delivery to tumors. Preclinical studies have shown that moderate aerobic exercise improves tumor vascular function and increases chemotherapy efficacy in mouse models, but the effect of exercise on human tumor vasculature has not yet been determined. Here, we demonstrate that exercise remodels the tumor vasculature, accelerates the regression, and delays the regrowth of pancreatic ductal adenocarcinoma in a patient-derived xenograft mouse model treated with gemcitabine. By evaluating pancreatic adenocarcinoma specimens from patients treated with preoperative chemotherapy or chemoradiation therapy, we also demonstrate for the first time that tumor vascular remodeling occurs in association with exercise in humans. Future studies will evaluate whether exercise-induced vascular remodeling improves gemcitabine or other chemotherapy efficacy in patients, as this study evaluated only changes in tumor vascular structure.

## Introduction

Physical activity as a component of care for cancer patients is well accepted as a method to treat fatigue, fitness loss, and the psychosocial side effects of cancer treatment^1–3^. In addition, pre-clinical evidence suggests that exercise is useful as an adjuvant to chemotherapy by improving chemotherapy delivery and anti-tumor efficacy. We and others have demonstrated that moderate aerobic exercise remodels tumor vasculature to improve blood delivery, and thus drug delivery, to prostate, breast, and pancreas tumors in mouse models^4–6^.

Pancreas ductal adenocarcinoma have a dense stromal component that compresses vasculature, stunts blood vessel growth, and reduces blood vessel function, preventing the effective delivery of drugs to the tumor cells. Therefore, one possible method for improving outcomes for patients receiving standard chemotherapy is to improve the vasculature within the tumor. In one study, the combination of gemcitabine plus treadmill walking inhibited the growth of a pancreatic ductal adenocarcinoma cell line subcutaneously injected into mice significantly more than gemcitabine alone. This greater inhibition depended on vascular remodeling after exercise, which correlated with more chemotherapy delivery to tumors^6^. When vascular remodeling was prevented pharmacologically or genetically, the improved efficacy afforded by exercise was lost, indicating a direct relationship between improved vasculature and improved chemotherapy efficacy.

Although the ability of moderate-intensity aerobic exercise to remodel tumor vasculature in animal models is well established, the exercise intensity or duration necessary to induce vascular remodeling in patients is unclear. It is also unclear whether human tumor vasculature will remodel in response to exercise, as predicted by animal models. To obtain the maximal benefit of exercise for patients, it is important to understand whether exercise can improve tumor vascular function. In this study, we used a clinically relevant pancreatic ductal adenocarcinoma patient-derived xenograft (PDX) to demonstrate that exercise combined with gemcitabine treatment significantly increases gemcitabine efficacy against the primary tumor and significantly prolongs the time to relapse. We recently completed a prospective clinical trial demonstrating that patients with pancreatic cancer who receive chemotherapy and chemoradiation therapy before surgery can engage in levels of home-based physical activity that approach the physical activity recommendations for cancer survivors^7–9^. We now show that the vasculature of tumors from patients who participated in that study is remodeled, as predicted by mouse models, and is different from the vasculature in tumors from control patients. This is the first clinical demonstration that an exercise intervention achievable by patients can directly impact the biology of the tumor, specifically by increasing the tumor vasculature.

## Results

### Exercise improves gemcitabine efficacy in a pancreatic cancer PDX model

**Figure 1:**
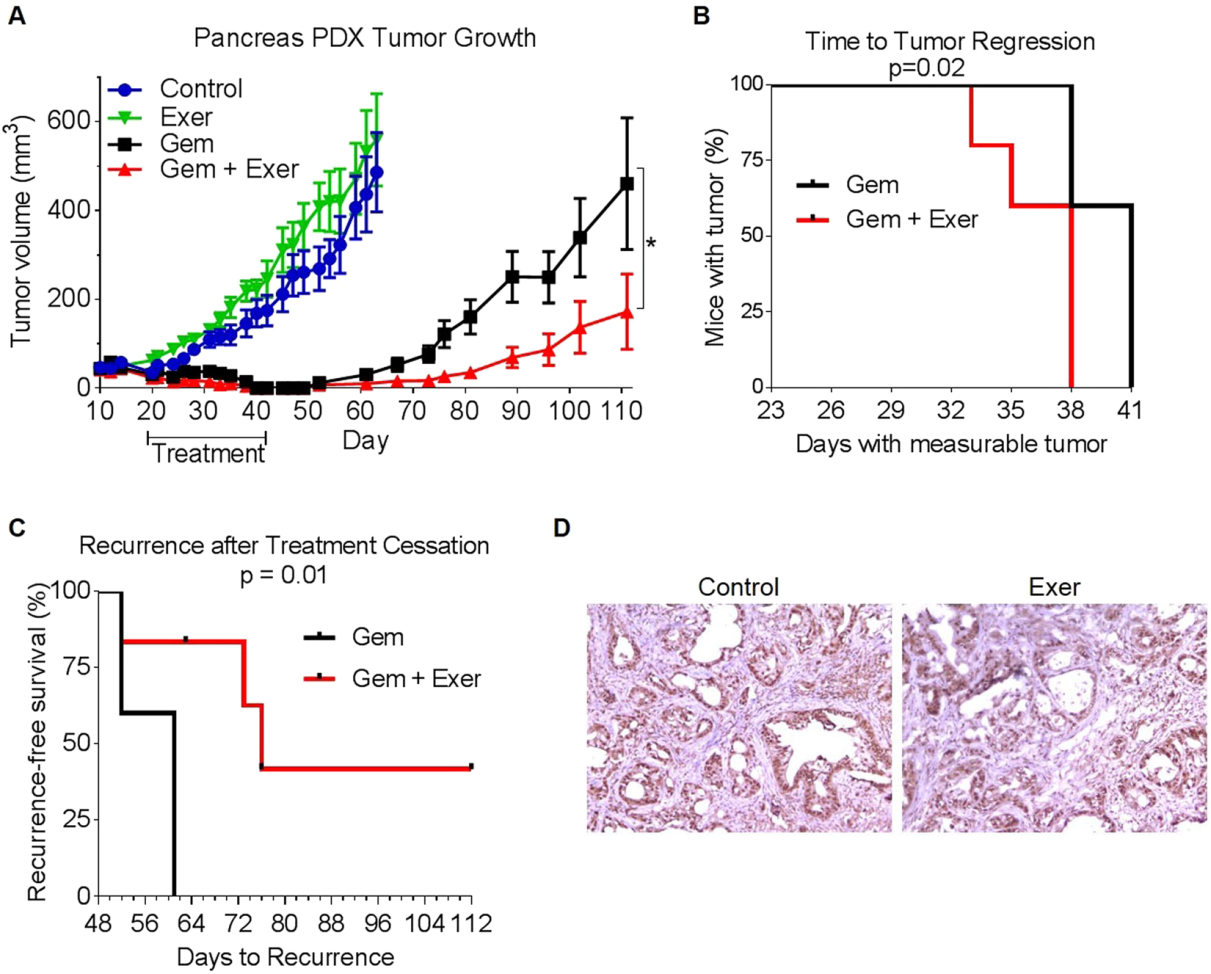
Exercise improves gemcitabine efficacy and delays regrowth. (**A**) Tumor volumes for control mice (blue), exercised mice (green), gemcitabine-treated mice (black), and gemcitabine-treated exercised mice (red). Data are expressed as means ± standard error of the mean. *p = 0.03. (**B**) The time to tumor regression for the mice treated with gemcitabine alone (Gem) was significantly longer than that for the mice treated with gemcitabine plus exercise (Gem + Exer; p = 0.02). (**C**) The time to regrowth for the mice treated with gemcitabine alone (Gem) was significantly shorter than that for the mice treated with gemcitabine plus exercise (Gem + Exer; p = 0.01). (**D**) Representative images of immunohistochemistry for ENT1 (brown) on PDX tumors from control and exercised mice. No difference in ENT1 was observed between tumors from control or exercise groups.

The effect of exercise on PDX tumors has not been previously evaluated. We thus used mice bearing subcutaneous pancreatic ductal adenocarcinoma-derived PDX tumors. The close similarity in histology between the PDX tumors retrieved from mice at the end of the experiment and human pancreatic ductal adenocarcinoma tumors was confirmed by a pathologist (H.W.). Mice bearing PDX tumors were treated with moderate treadmill exercise, gemcitabine, or a combination of exercise plus gemcitabine or with phosphate-buffered saline (PBS; control). Exercise alone had no effect on tumor growth relative to control; however, gemcitabine alone or exercise plus gemcitabine caused tumor regression followed by a period of no detectable tumor and then tumor regrowth (Fig. 1A). The time to tumor regression (no palpable tumor) for mice receiving the combination of exercise plus gemcitabine was significantly shorter than that for mice receiving gemcitabine alone (p = 0.02, Fig. 1B).

Gemcitabine and exercise were stopped after 4 weeks. At that time, one-third of the mice treated with gemcitabine and two-thirds of mice treated with exercise plus gemcitabine had no palpable tumors. However, these mice had tumor regrowth 12−74 days later. The mice treated with gemcitabine alone had a significantly shorter time to recurrence than did those treated with exercise plus gemcitabine (p = 0.01, Fig. 1C). The increased efficacy of gemcitabine against PDX tumors in the presence of exercise suggested that exercise may change the expression of the gemcitabine transporter hENT1^10^ on tumor cells. However, there was no difference in hENT1 expression between tumors from control or exercised mice as determined immunohistochemically (Fig. 1D).

**Figure 2:**
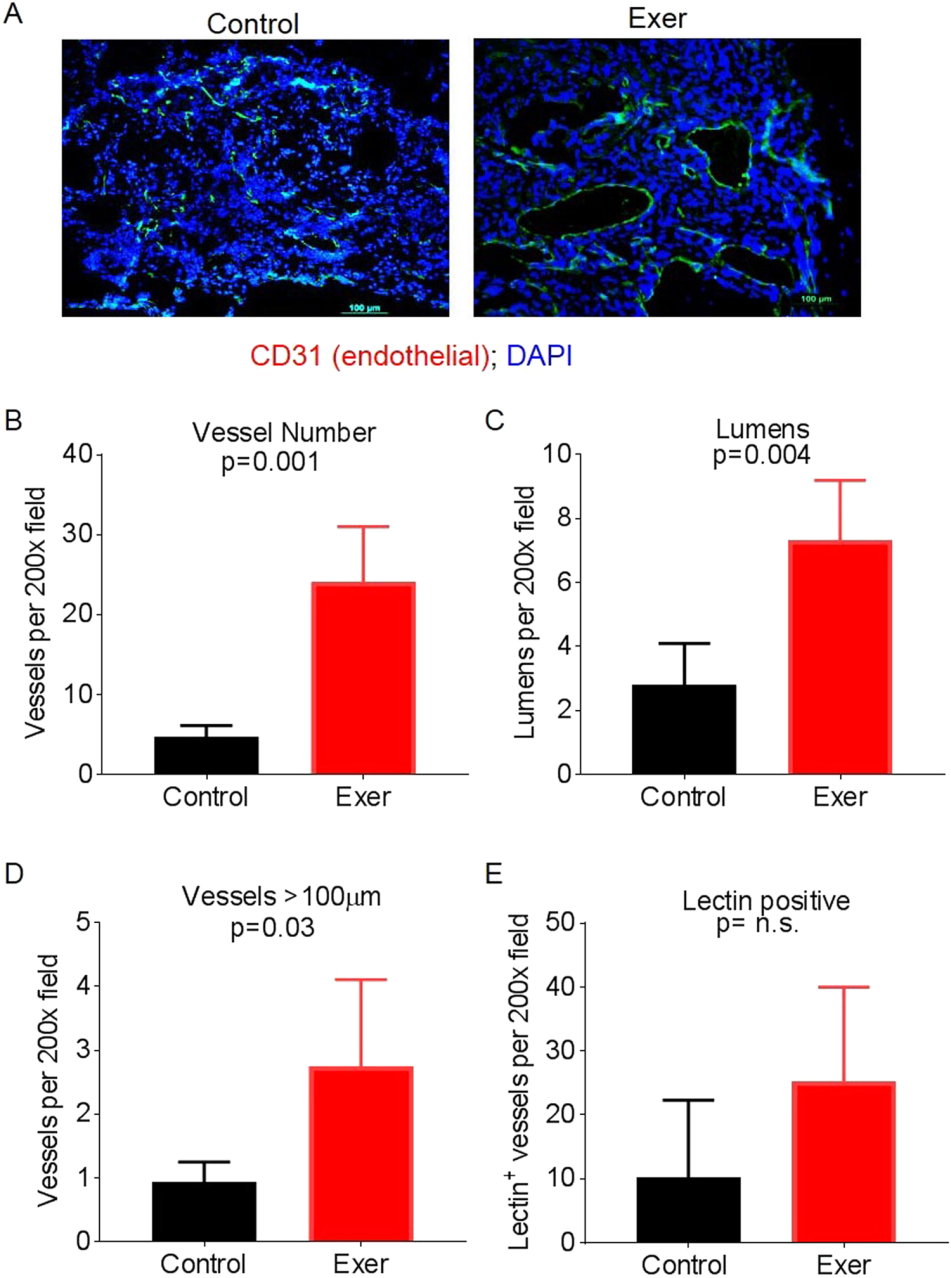
Exercise induces vascular remodeling in PDX tumors. (**A**) Immunofluorescent staining for endothelial cells (green) and nuclei (blue). (**B**−**E**) Exercise changed the number and structure of blood vessels. The average numbers of vessels (**B**), lumens (**C**), vessels >100 μm (**D)**, and lectin-positive (functional) vessels (**E**) per 200x field were quantified. Graphs show means ± standard deviations for 5 fields per tumor (n = 5 per group).

Thus, we evaluated the possibility of changed delivery of gemcitabine to tumors from exercised mice due to changes in tumor vasculature. Examination of immunofluorescent staining for CD31^+^ endothelial cells within the PDX tumors revealed that tumors from exercised mice had more vascularity than tumors from control mice did (Fig. 2A). Indeed, compared with tumors from control mice, tumors from exercised mice had significantly more vessels per 200x field (mean 24.0 vs 4.6, p = 0.001), open lumens (mean 7.3 vs 2.8, p = 0.004), and vessels longer than 200 μm (mean 2.75 vs 0.90, p = 0.03; Fig. 2B–D), indicating that the tumors from exercised mice underwent vascular remodeling. Tumors from exercised mice also had a higher percentage of functional vessels as assessed by lectin perfusion, but this difference was not statistically significant (Fig. 2E). Vasculature was not evaluated in mice that had received gemcitabine with or without exercise; because 74 days had elapsed between the final session of exercise and the time of euthanasia, exercise-induced changes in tumor vasculature were likely to be masked by vascular regrowth in those 74 days.

### Exercise changes the tumor vascular structure in pancreatic cancer patients

**Figure 3:**
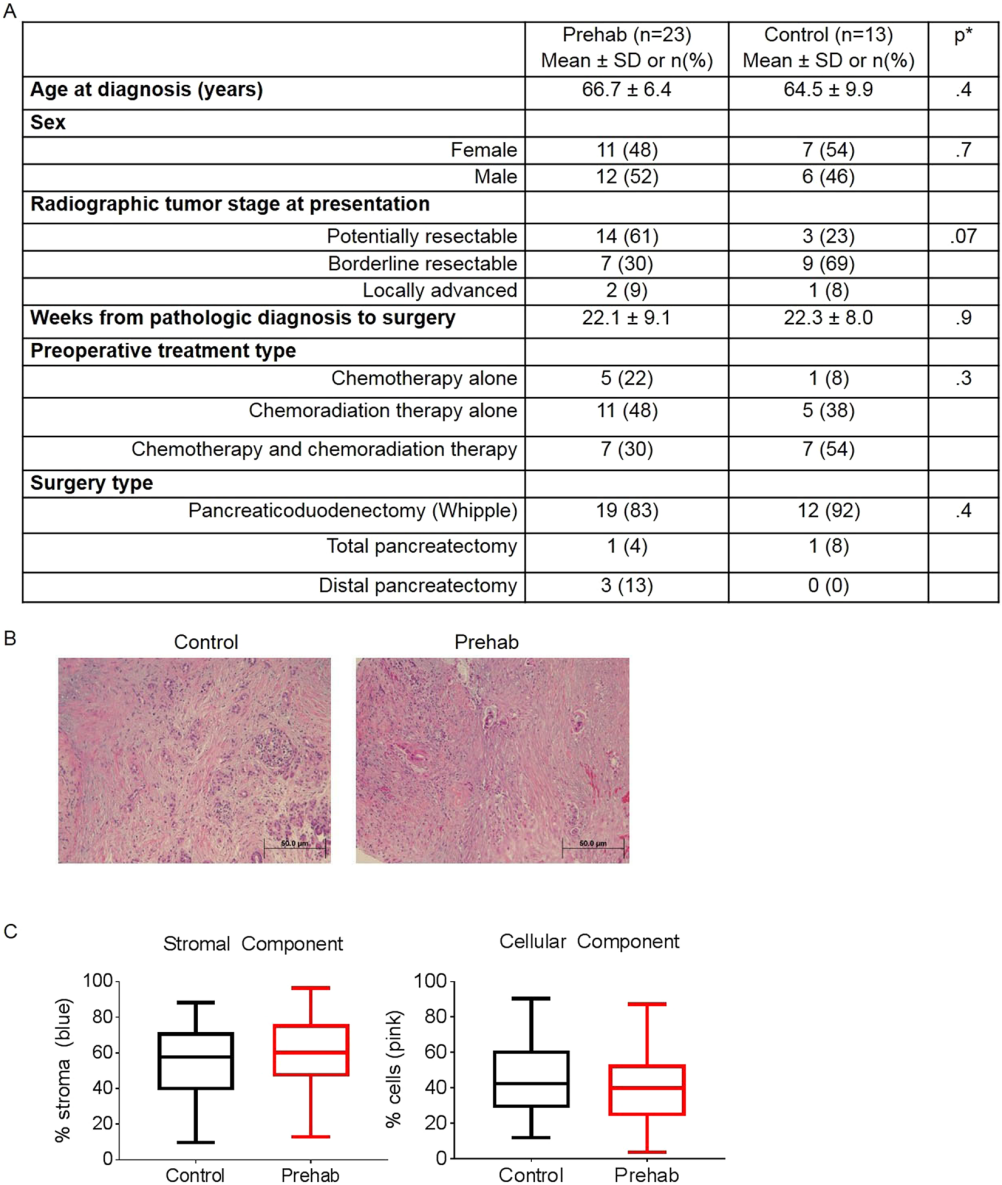
Exercise does not change tumor stroma or inflammation. (**A−C**) Prehab and control groups showed no difference in patient demographics or disease stage as assessed by a pathologist (**A**), tumor histology as assessed by H&E staining (**B**), or tumor stromal or cellular components as assessed by Masson trichrome staining (**C**).

Although the impact of exercise on tumor vasculature in mouse models has been reported^6^, no one has yet demonstrated whether exercise can impact tumor vasculature in humans. We recently completed a study of 70 patients with potentially resectable pancreatic cancer who were prescribed exercise concurrently with preoperative chemotherapy and/or chemoradiation^7,8^. Participants were prescribed at least 120 minutes of moderate-intensity, home-based exercise (60 minutes of aerobic exercise and 60 minutes of strengthening per week) and were instructed to record minutes of physical activity in daily exercise logs. Of the 70 patients enrolled, 33 (47%) underwent resection of their primary tumor; tumor tissue and activity data were available for 23 of those 33 patients. These 23 patients (comprising a “prehab” group) were enrolled in the exercise program for a mean of 14.87 ± 6.46 weeks and reported an average of 172.6 ± 80.0 minutes per week of aerobic and strengthening exercise prior to surgery. These patients’ tumor samples were compared with 13 historical control tumors obtained from patients who underwent surgery at MD Anderson Cancer Center following chemotherapy or chemoradiation therapy and who had not participated in a prescribed exercise program. Patient demographics, disease stage, treatment, and time from pathological diagnosis to surgery were not significantly different between the prehab and control groups (Fig. 3A).

**Figure 4:**
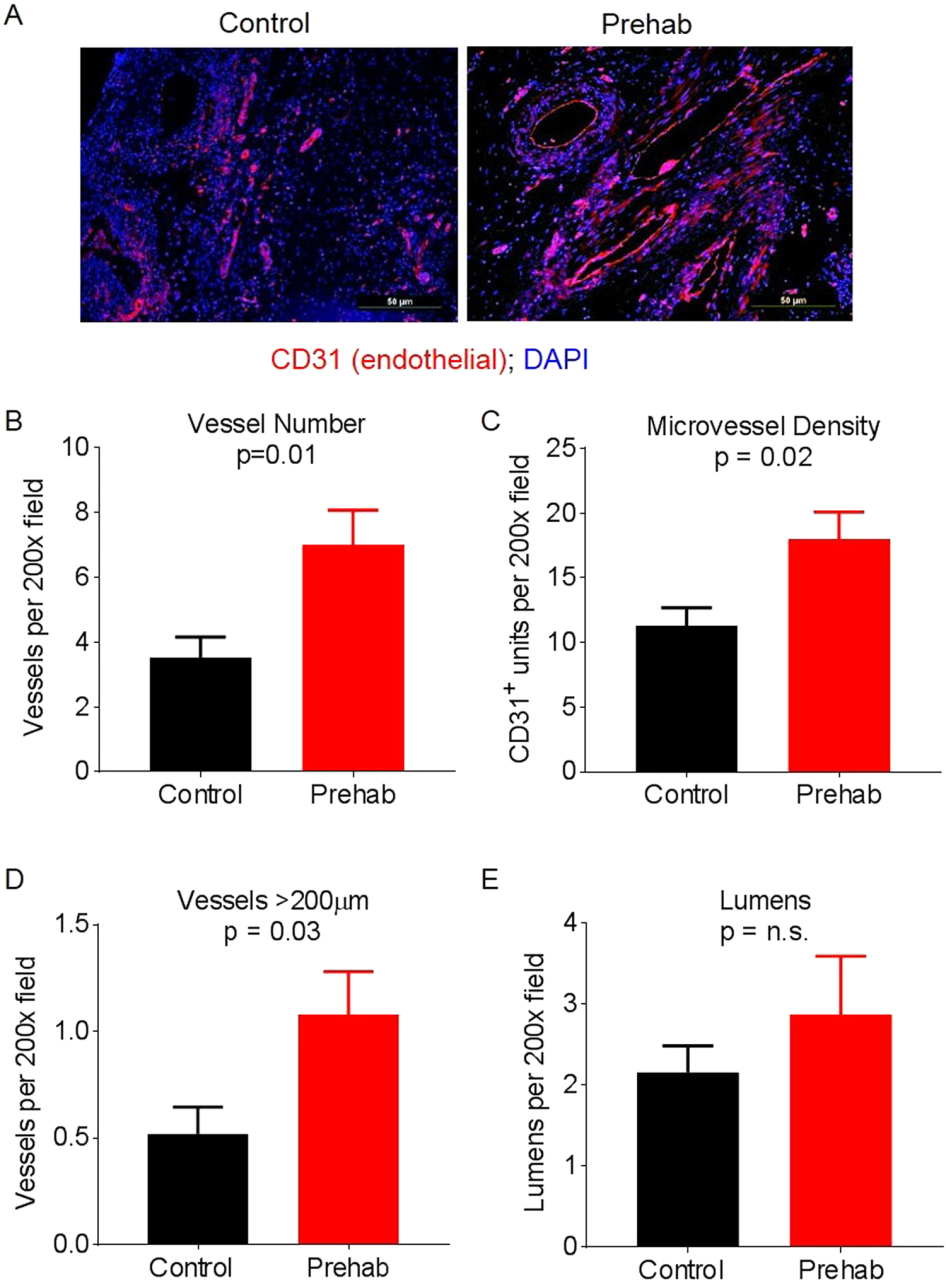
Exercise induces vascular remodeling in pancreas tumors from patients. (**A**) Immunofluorescent microscopy was used to evaluate surgical specimens from patients who participated in the exercise intervention (“prehab”) and those from historical controls for changes in vascular structure. Red indicates CD31 staining (endothelial cells), and blue indicates DAPI staining (nuclei). (**B−E**) Five random sections per tumor were evaluated to obtain one value per tumor for the number of total vessels (**B**), microvessel density (**C**), number of elongated vessels (**D**), and number of visible open lumens (**E**). Graphs show the means ± standard error of the mean for 15 samples from historical controls and 23 samples from prehab patients.

Tumors from control group patients and tumors from prehab group patients had no differences in tumor (T) stage, lymph node status (N stage), AJCC stage (8^th^ edition) or tumor regression grade (percentage of viable tumor; Table S1). There were no other differences noted upon assessment of hematoxylin & eosin (H&E) stained slides by a pathologist blinded to whether the tissue was from historical controls or patients who participated in prehab exercise (Fig. 3B), or in stromal and cellular components as quantified by Masson trichrome staining (Fig. 3C). There were also no significant differences between the groups in the degree of fibrosis or inflammation as determined by the pathologist (data not shown). However, the vasculature in tumors from prehab patients was remodeled, as predicted by mouse models. Immunofluorescent staining for CD31^+^ endothelium revealed a striking difference between the tumor vasculature in the prehab group and that in the control group (Fig. 4A). Compared with control group tumors, prehab group tumors had twice as many total vessels per field (7 ± 5.07 vs 3.53 ± 2.44, p = 0.01, Fig. 4B) and significantly higher microvessel density (17.97 ± 9.96 vs 11.27 ± 5.51 CD31^+^ units per field, p = 0.02, Fig. 4C). Further, tumors from prehab patients had significantly more elongated vessels (p = 0.03, Fig. 4D) and more open vessel lumens (Fig. 4E).

## Discussion

In this study, we demonstrated that moderate treadmill exercise remodels tumor vasculature and increases the efficacy of gemcitabine against PDX pancreatic tumors. We also demonstrated that exercise delays the regrowth of such pancreatic tumors, as mice that received exercise as an adjuvant to gemcitabine had a significantly longer time to tumor regrowth than those receiving gemcitabine alone did. Finally, we showed that an attainable, home-based exercise prescription for patients with potentially resectable pancreatic cancer who are undergoing chemotherapy or chemoradiation therapy may be sufficient to remodel tumor vasculature, as predicted by studies in mouse models. This study is the first to provide evidence that, in addition to improving quality of life and physical function, exercise directly impacts tumor biology in patients undergoing cancer treatment and therefore may prolong survival.

Several studies have demonstrated that aerobic exercise alters the blood flow to tumors in mice and rats^4,5^. In addition to finding an acute increase in blood delivery to tumors during exercise, we and others have demonstrated that exercise causes a change in the structure and function of tumor vasculature lasting several days, which can be exploited to improve chemotherapy delivery and efficacy. In the present study, we improved upon those findings using a PDX model that more closely models human pancreatic ductal adenocarcinoma. Hypovascularity of pancreatic ductal adenocarcinoma, caused in part by dense stroma compressing the vasculature, impedes the delivery of chemotherapy to tumor cells. Our data demonstrating increased tumor vasculature and a significantly increased time to recurrence regrowth after moderate-intensity aerobic exercise, in the absence of changes in hENT-1 expression by tumor cells, suggests that the improved chemotherapy delivery enabled by exercise may reduce recurrence and thus warrants further investigation.

Whether the numerous animal studies demonstrating that exercise is an effective adjuvant to chemotherapy accurately predict the usefulness of exercise for patients is unclear, in part because it is unclear whether human tumor microenvironment components such as endothelium will respond to exercise, as occurs in mice. We previously demonstrated that pancreatic cancer patients undergoing preoperative therapy can achieve modest, home-based exercise recommendations^7,8^. Here, we report for the first time that a feasible exercise intervention influences tumor vasculature remodeling in patients, as predicted by animal studies. We found that, compared with historical controls, patients who participated in an exercise intervention during preoperative chemotherapy or chemoradiation therapy had strikingly different tumor vasculature, including significantly higher microvessel density and more elongated blood vessels. While we did not evaluate the duration of vascular remodeling in this study, tumors from several patients who did not exercise the day prior to surgery still had evidence of vascular remodeling, suggesting that the effect lasts at least 24 hours. Future studies evaluating the duration of vascular remodeling would be interesting and worthwhile. This exciting finding suggests that exercise changes vascular biology within the tumor and may be a low-cost, low-risk method for improving chemotherapy delivery and thus efficacy.

Limitations of our study include the fact that patients were not followed for short-term or long-term outcomes, thus tumor vascular structure cannot be correlated to clinically relevant outcomes including tumor response or survival. Additional limitations include the fact that exercise and physical activity habits prior to enrollment in our study may impact tumor biology but were not measured and cannot be correlated to our findings. Our future studies will test this possibility by comparing the vascular structures and clinical outcomes of patients who participate in exercise during preoperative therapy with those of patients who do not. Finally, this study did not evaluate whether chemotherapy was more effective in patients who exercised. Future studies will be needed to determine whether exercise-induced tumor vascular remodeling can improve efficacy of chemotherapy in patients.

## Methods

### PDX tumor model

All animal experiments were approved by the Institutional Animal Care and Use Committee of MD Anderson Cancer Center, and were performed in accordance with approved protocols. Pancreatic ductal adenocarcinoma (PDAC) tumor tissue obtained from a surgical resection was used to establish a PDX. Our work used an established PDX transferred from a host mouse by subcutaneous implantation into the flank of one male nude mouse (mice purchased from MD Anderson Cancer Center radiation oncology animal breeding resource). When the tumor reached approximately 500 mm^3^, the mouse was euthanized by carbon dioxide and the tumor was resected, divided, and implanted into 5 female nude mice; the tumor was propagated in this manner two further passages to obtain enough mice to perform each experiment. For each experiment, when implanted tumors reached approximately 50 mm^3^, the mice were divided into 4 treatment groups of 5–7 mice each. The treatment groups received intraperitoneal injections of PBS (3 times per week; control), exercise only (treadmill running, 12 meters/minute, 45 minutes/day, 5 days/week), intraperitoneal injections of gemcitabine only (15 mg/kg, 3 times per week,), or a combination of exercise plus gemcitabine. After 4 weeks, all treatment was stopped, and mice were monitored for tumor regrowth by twice weekly palpation and visual inspection.

### Exercise intervention and patient samples

All aspects of the clinical study were approved by the Institutional Review Board of MD Anderson Cancer Center (protocol #2014-0702 and PA16-0249; ClinicalTrials.gov Identifier: NCT02295956 registration date 20/11/2014). All methods were performed in accordance with relevant guidelines and regulations, including informed consent, as approved by the Institutional Review Board. The clinical trial has been described in detail^7,8^ and is shown in Supplemental Information. Briefly, participants were prescribed at least 60 minutes of aerobic exercise per week and 60 minutes of strengthening exercises per week and used daily logs to record their activity. A subset of these patients used accelerometers to confirm weekly minutes of physical activity. Patients who participated in the exercise intervention, referred to as “prehab” were compared to historical controls. Historical controls were not different than prehab patients on the basis of age, sex, disease stage, treatment, time from pathological diagnosis to surgery,tumor (T) stage, lymph node status (N stage), AJCC stage (8^th^ edition) or tumor regression grade (percentage of viable tumor; Table S1).

Of 70 patients who enrolled, only 65 participated (turned in exercise logs). Of those 65, only 34 patients went to surgery. Those who did not undergo surgery had disease progression while on treatment. Of the 34 patients who completed exercise logs and underwent surgery, excess tissue for research purposes was available for 23 patients. Sections of tumors from prehab or control patients who underwent surgery and for whom excess tissue was available after pathological evaluation were subjected to immunohistochemical analyses.

### Immunohistochemical analysis

PDX-bearing mice were injected with Alexa594-labeled tomato lectin (100 μg/kg; Invitrogen) and then euthanized by carbon dioxide. Tumors were dissected and immediately flash-frozen in optimal cutting temperature reagent. The tumors were then sectioned, fixed in ice-cold acetone for 10 minutes, washed with PBS, and incubated with rat anti-mouse CD31 antibody (BD Pharmingen 553370) diluted in Tween 0.3%/1% bovine serum albumin/5% normal goat serum overnight at 4 °C. The slides were then washed in PBS, incubated with AlexaFlour488-conjugated secondary antibody (Invitrogen) for 1 hour at room temperature, washed in PBS three more times, mounted in Fluoro-Gel II with DAPI (Electron Microscopy Sciences), and coverslipped. For hENT1, samples were evaluated as described^10^ with the only change being that samples frozen in OCT were fixed in 4% PFA for 30 minutes prior to antigen retrieval.

Slides of human tumor sections were de-paraffinized with xylene and ethanol. The slides were then incubated in buffer containing 20 μg/mL Tris, EDTA, and Tween20 (pH 9.0) at 37 °C for 20 minutes for antigen retrieval, washed in Tris-buffered saline (TBS), and incubated with anti−human CD31 antibody (Abcam AB28364) overnight at 4 °C. The slides were then washed in TBS, incubated with AlexaFluor594-conjugated secondary antibody (Invitrogen) for 1 hour at room temperature, washed in TBS three more times, mounted in Fluoro-Gel II with DAPI (Electron Microscopy Sciences), and coverslipped. Masson trichrome staining and H&E staining were performed by MD Anderson Cancer Center’s Research Histology Core Laboratory and analyzed by a pathologist who was blinded to whether the patient participated in exercise.

Images were captured with a Nikon Eclipse upright microscope imaging system (Leica Microsystems) and analyzed using SimplePCI6 (Legacy) or Adobe Photoshop software. For the quantification of tumor vasculature, the areas of CD31-positive structures (microvessel density) were measured and the vessels, vessels longer than 200 μm, and with visible lumens were counted in 5 random 200X magnification fields of each slide.

### Statistics

Among the mouse treatment groups, times to regression and times to regrowth were compared using the Kaplan-Meier method, and tumor volumes were compared using 2-way ANOVA. Vasculature was compared by analyzing 5 random sections per tumor to create one value per tumor, and then the groups were compared using the Student t-test.

Summary statistics were used to describe patients’ prehabilitation status; weekly minutes of aerobic exercise and strengthening exercise; as well as microvessel density, numbers of blood vessels, open lumens, and vessels >200 μm and degree of inflammation and fibrosis. The chi-square test or Fisher exact test, whichever more appropriate, was used to compare categorical variables between the prehab and control groups. Wilcoxon rank-sum test was used to compare continuous variables between these two groups.

All computations were carried out in SAS 9.4 (SAS Institute Inc., Cary, NC, USA). P values were considered significant if they were ≤0.05.

## Supplementary information

Supplemental Table 1.Disease Characteristics

Supplemental Information 1. Clinical protocols
