# Supplementary material for: Exercise during preoperative therapy increases tumor vascularity in pancreatic tumor patients
Source: Sci Rep. 2019 Sep 27;9:13966. doi: 10.1038/s41598-019-49582-3 (PMC6765012; doi:10.1038/s41598-019-49582-3)
Supplement: Supplementary file 1 — Supplemental Table 1.Disease Characteristics [file 41598_2019_49582_MOESM1_ESM.docx]

**Exercise during preoperative therapy increases tumor vascularity in pancreatic tumor patients**

Claudia Alvarez Florez, Ana Carolina Ferreira Cardoso, Nathan Parker, An Ngo-Huang, Maria Q. Petzel, Michael P. Kim, David Fogelman, Salvador Gabriel Romero, Huamin Wang, Minjeong Park, Matthew H.G. Katz, and Keri L. Schadler

**SUPPLEMENTAL TABLE**

**
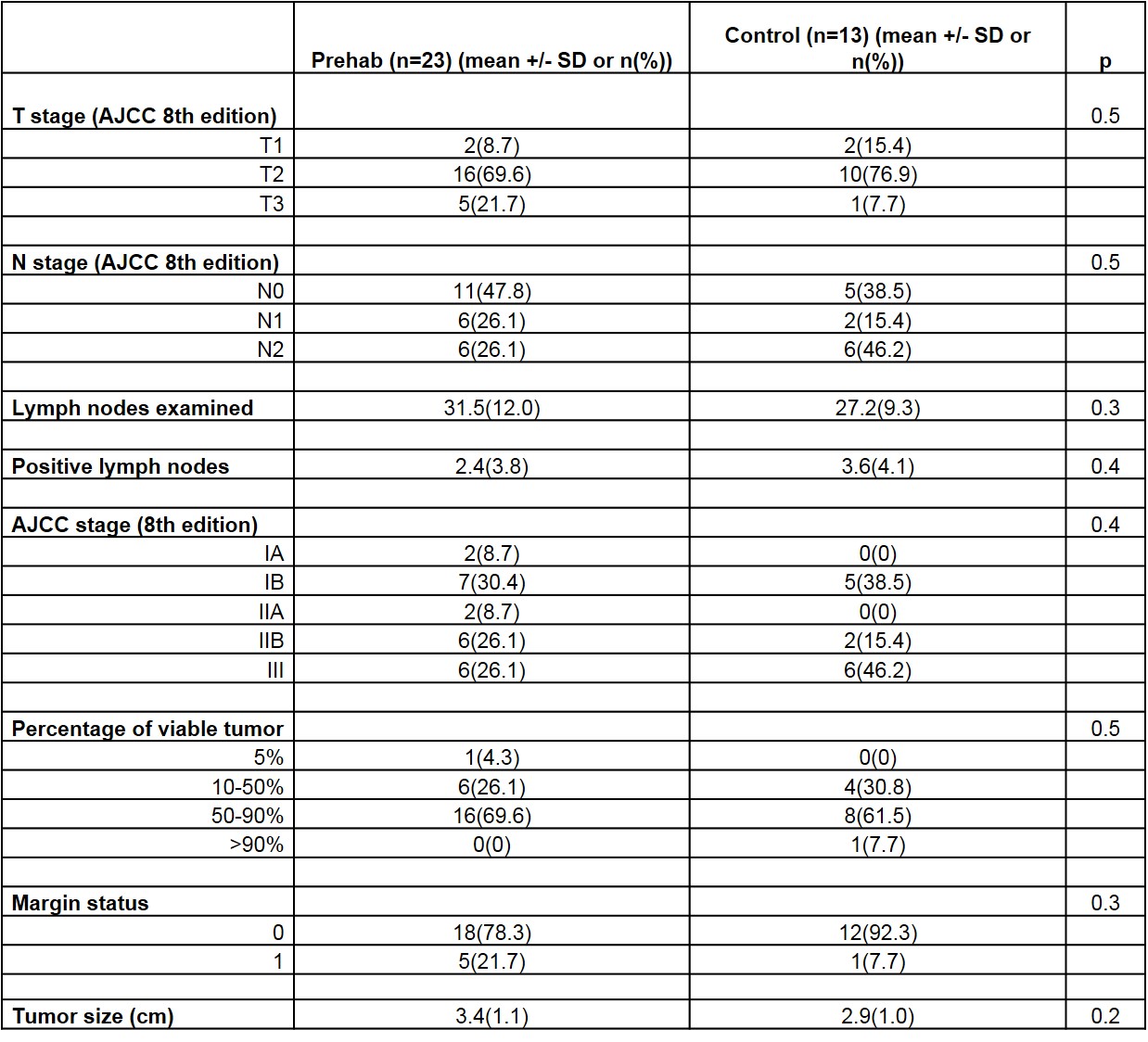
**

**Supplemental Table 1. Disease characteristics in patient cohorts.** Patients who participated in the exercise program (“prehab”) were not different from historical controls in T stage, N stage, Lymph nodes examined, Positive lymph nodes, AJCC stage, Percentage of viable tumor, margin status, or tumor size.
