# Supplementary material for: Exercise during preoperative therapy increases tumor vascularity in pancreatic tumor patients
Source: Sci Rep. 2019 Sep 27;9:13966. doi: 10.1038/s41598-019-49582-3 (PMC6765012; doi:10.1038/s41598-019-49582-3)
Supplement: Supplementary file 2 — Supplemental Information 1. Clinical protocols [file 41598_2019_49582_MOESM2_ESM.pdf]

# Exercise during preoperative therapy increases tumor vascularity in pancreatic tumor patients

Claudia Alvarez Florez[1], Ana Carolina Ferreira Cardoso[1,8], Nathan Parker[2,3], An Ngo-Huang[4], Maria Q. Petzel[2], Michael P. Kim[2], David Fogelman[5], Salvador Gabriel Romero[1], Huamin Wang[6], Minjeong Park[7], Matthew H.G. Katz[2], and Keri L. Schadler[1]

Accompanying protocol PA16-0249

# *TITLE: Retrospective study of the exercise effect on pancreatic ductal adenocarcinoma tumor vasculature and immune cell infiltrate*

Study Chair: Keri Schadler, PhD
Study co-Chairs: Matthew Katz, MD; An Ngo-Huang, MD; David Fogelman, MD
Department: Surgical Oncology
Phone: 713-745-2168
Unit: 1484

## Table of Contents

1.0 Objectives ........ 3
2.0 Rationale ........ 3
3.0 Eligibility of Subjects ........ 4
4.0 Research Plan and Methods ........ 4
5.0 Statistics and Justification of Sample Size ........ 5
6.0 Request for Waiver of Informed Consent ........ 5
7.0 Data Confidentiality: ........ 6
8.0 References ........ 7

## 1.0 Objectives

**1.** Determine whether the vascular phenotype (average vessel length, number of open lumens, number of endothelial cell sprouts, pericyte coverage of vessels) of pancreatic adenocarcinoma is associated with patient pre-surgery physical activity level.

**2.** Determine whether the number of infiltrating immune cells in pancreatic adenocarcinoma is associated with pre-surgery physical activity level.

**3.** Determine whether serum levels of angiogenic factors are associated with patient physical activity level.

## 2.0 Rationale

Chemotherapy and/or radiation therapy is administered to patients with potentially curable pancreatic adenocarcinoma (PDAC) prior to pancreatectomy (1, 2). Chemotherapy efficacy is dependent on chemotherapy access to the tumor cells, via tumor vasculature. However, PDAC tumors are poorly perfused with compressed, dysfunctional tumor vasculature that ineffectively delivers chemotherapy to the tumor (3).

In mouse models of PDAC, we demonstrated that moderate aerobic exercise changed the tumor vascular phenotype. Tumors from exercised mice had longer vessels with more open lumens, and a 24% increase in the percentage of functional blood vessels (fig 1). Importantly, the combination of exercise and gemcitabine correlated with better delivery of chemotherapy to PDAC tumors and with significantly better inhibition of tumor growth than gemcitabine alone (fig 2). In our mouse models, we identified Thrombospondin-1 and Sphingosine-1-Phosphate as circulating angiogenic factors that are responsible for improved tumor vascular function due to exercise. Further, preclinical evidence suggests that exercise may increase the number of cytotoxic NK cells recruited a tumor, further bolstering anti-tumor effect (4). If our findings in mice accurately represent human tumors, this would be compelling evidence to support exercise as an adjuvant to chemotherapy. We therefore propose a retrospective lab study to evaluate vascular phenotype and immune cell infiltration in PDAC samples, and to correlate these findings with patient physical activity levels.

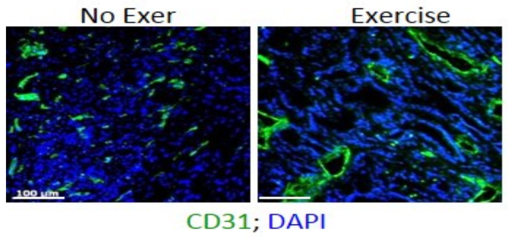


**Fig 1. Vascular Changes in PDAC tumors from exercised mice.** PDAC-tumor bearing mice performed moderate treadmill running 5 days/week for 2 weeks. Tumor vasculature was evaluated by immunohistochemistry for CD31, and the average microvessel density, number of open lumens, vessel length, and percent functional vessels were quantified.

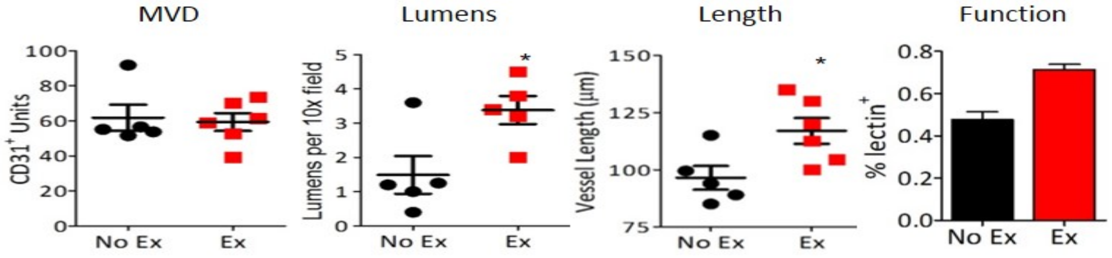

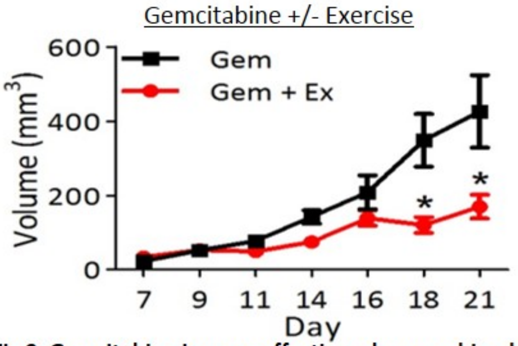


**Fig 2. Gemcitabine is more effective when combined with exercise.** PDAC-tumor bearing mice were treated with 15mg/kg gemcitabine 3x weekly, and performed moderate treadmill running 5 days/week for 2 weeks or were in the control group. Tumor volume over time is shown, n=5 per group, *p<0.05.

## 3.0 Eligibility of Subjects

1. Patient is enrolled on 2014-0702.
2. Patient has agreed to banking of samples and use of samples in future research, and has samples available for analysis through Protocol PA11-0670.

## 4.0 Research Plan and Methods

Tissue Source: For PDAC tumor tissue, sections from surgical specimens collected under protocol Lab00-396 (PSC), or from the MD Anderson tissue bank will be used. For serum samples, blood collected under PA11-0670 will be used.

Tissue Sample Number: Samples will be divided into “control, unknown physical activity” or “documented preoperative physical activity” groups. For control, unknown physical activity group, 30 tumor samples and 30 blood samples will be evaluated. Samples will be classified as “documented preoperative physical activity” if the samples were obtained from patients who participated in the Preoperative Rehabilitation during neoadjuvant therapy for pancreatic cancer (2014-0702) study and therefore detailed documentation of the patients’ physical activity can be obtained. For this group, 60 tumor samples and 60 blood samples will be evaluated.

Immunohistochemistry: Standard immunohistochemistry staining protocols will be used to stain tumor sections for CD31 or CD34 (endothelial cells), a-Smooth Muscle Actin (pericytes/smooth muscle cells), CD8, CD4 (T cells), or CD94 (NK cells). After staining, 5 random 10x magnification fields per tumor will be photographed, and the following will be quantified and averaged to obtain one value per patient: microvessel density, number of blood vessels per field, average vessel length, number of open lumens, number of endothelial cell sprouts, number of $CD8^+$ cells, number of $CD4^+$ cells, number of $CD94^+$ NK cells.

Serum Thrombospondin-1 and Sphingosine-1-Phosphate: Serum will be collected and batch processed, 10 samples at a time. Commercially available ELISAs for human Thrombospondin-1 and Sphingosine-1-Phosphate.

Association with Patient Characteristics: Patient medical records will be used to classify each patient as either: Unknown Physical Activity, 0-60 minutes weekly Physical Activity, 60-120 minutes weekly Physical Activity, or >120 minutes weekly Physical Activity. Values for all measured variables will then be grouped by physical activity levels and compared for associations.

## 5.0 Statistics and Justification of Sample Size

The major objective of this study is to correlate pre-surgery activity level with vascular phenotype and infiltrating immune cells (i.e. CD8+, CD4+ and CD94+ NK cells) in pancreatic adenocarcinoma, as well as to correlate pre-surgery activity level with serum level of angiogenic factors Thrombospondin-1 and Sphingosine-1-Phosphate. Vascular phenotype outcomes include microvessel density, number of blood vessels per field, average vessel length, number of open lumens.

Tumor and blood sample from two groups of patients, i.e. control, unknown physical activity or documented preoperative physical activity, will be studied. 30 tumor samples and 30 blood samples will be evaluated for patients with unknown pre-surgery physical activity, while 60 tumor samples and 60 blood samples will be evaluated for patients with documented preoperative physical activity, and patients in this group will be classified based on their duration of weekly physical activity as three groups: 0-60 minutes, 60-120 minutes and > 120 minutes, with an expected proportions of 25%, 50% and 25% for each group, respectively. We will use ANOVA with pairwise comparisons to compare vascular phenotype outcomes, number of infiltrating immune cells and angiogentic factors between groups with different activity levels, and will adjust for multiple tests using Tukey's method. Or we will use non-parametric alternatives Kruskal-Wallis test or Wilcoxon rank sum test if the assumption of normality is not satisfied. Sample size justification was calculated based on comparison of one outcome between any two groups with different activity levels, using a two-sided two sample t test at a significance level of 0.008 (Bonferroni method adjusted). A sample size of 15 samples per group will have 80% power to detect an effect size (i.e. the expected difference in means divided by the common standard deviation) of 1.362 in the measured outcome between two comparison groups. A sample size of 30 samples in one group and 15 in the other group will have 80% power to detect an effect size of 1.152. While a sample size of 30 samples per group will have 80% power to detect an effect size of 0.93. With sample size increases, 80% power will be able to detect a smaller effect size. In addition, we will also fit multivariate linear regression models to compare vascular phenotype outcomes, infiltrating immune cells and angiogenic factors among patient group with different levels of physical activity, adjusting for the effects of patient baseline characteristics. In the event that the normality assumption is not satisfied, data transformation will be performed.

## 6.0 Request for Waiver of Informed Consent

A Waiver of Informed Consent and a Waiver of Authorization is requested because this is a retrospective chart review that involves no diagnostic or therapeutic intervention, as well as no direct patient contact. Study staff are unable to obtain consent from study subjects because due to the international patient population of M. D. Anderson Cancer Center, it would be impractical to obtain informed consents from individual patients prior to beginning the protocol. Additionally, it would bias the study if it were restricted to only those patients that we were able to contact. It is not practical to conduct this research

without this waiver since the status of the patient is unknown, i.e. whether they are alive or deceased and it is difficult to trace the whereabouts of the patients. The following requests for waivers are attached to this protocol:

Request for Waiver of Informed Consent
Request for Waiver of Authorization to Use and Disclose Protected Health Information (PHI)

## 7.0 Data Confidentiality:

Data will only be available to the PI and people directly involved with the collection and analysis of data related to this project.

Collection of Identifiers: Identifiers (name, medical record number) will be collected but will be replaced by study numbers in the analytic file. The key linking these numbers will be retained in a locked file by the investigator designated personnel

Training of personnel: Only MDACC personnel designated by the Principal investigator will have access to study records. These personnel will be fully trained to maintain the patient health information confidential.

Data Storage: The PI and research staff will attempt to minimize risk through only storing information containing subject identifiers in locked file storage, on password-protected computers, and/or in password protected databases. In addition, access to patient identifiers will be limited to the minimum number of necessary research personnel, and only to those research personnel directly involved with obtaining patient information and assigning random study identifiers. Hard-copy information containing subject identifiers will be properly destroyed, using secured-information disposal sites and total-destruction shredding equipment to assure that subject privacy and confidentiality are protected. Keys containing information linking study subjects to personal identifiers will be maintained in locked storage and available ONLY to the PI and research personnel directly involved in creating random study identifiers. Information containing subject personal identifiers will not be removed from MD Anderson Cancer Center and will not be shared in publications or reports concerning this research study.

Data Sharing: Study data will not be shared with any individuals or entities that are not involved in the study.

Sample Management: Physical samples from this study will be obtained from protocol PA11-0670. Samples will be identified by a unique study identifier. PHI will be obtained in order to be able to match samples to data. The linking key between the sample identifier and the patient PHI will be retained by the PI on a secured, encrypted computer behind an institutionally approved firewall. Information from the PI Key will NOT be shared outside of

MD Anderson, but may be shared with study Co-PIs and research personnel who require it to facilitate study tests and assign test results.

Final disposition of study records: These data will be used only for this research study. Study data, paper records will be destroyed within 10 years after termination of the study.

## 8.0 References

1. Cooper AB, Holmes HM, des Bordes JKA, Fogelman D, Parker NH, Lee JE, Aloia TA, Vauthey J-N, Fleming JB, Katz MHG. Role of Neoadjuvant Therapy in the Multimodality Treatment of Older Patients with Pancreatic Cancer. Journal of the American College of Surgeons. 2014;219(1):111-20. doi: http://dx.doi.org/10.1016/j.jamcollsurg.2014.02.023.
2. Tzeng C-WD, Fleming JB, Lee JE, Xiao L, Pisters PWT, Vauthey J-N, Abdalla EK, Wolff RA, Varadhachary GR, Fogelman DR, Crane CH, Balachandran A, Katz MHG. Defined Clinical Classifications Are Associated with Outcome of Patients with Anatomically Resectable Pancreatic Adenocarcinoma Treated with Neoadjuvant Therapy. Annals of Surgical Oncology. 2012;19(6):2045-53. doi: 10.1245/s10434-011-2211-4.
3. Feig C, Gopinathan A, Neesse A, Chan DS, Cook N, Tuveson DA. The Pancreas Cancer Microenvironment. Clinical Cancer Research. 2012;18(16):4266-76. doi: 10.1158/1078-0432.ccr-11-3114.
4. Pedersen L, Idorn M, Olofsson Gitte H, Lauenborg B, Nookaew I, Hansen Rasmus H, Johannesen Helle H, Becker Jürgen C, Pedersen Katrine S, Dethlefsen C, Nielsen J, Gehl J, Pedersen Bente K, thor Straten P, Hojman P. Voluntary Running Suppresses Tumor Growth through Epinephrine- and IL-6-Dependent NK Cell Mobilization and Redistribution. Cell Metabolism. doi: http://dx.doi.org/10.1016/j.cmet.2016.01.011.

Exercise during preoperative therapy increases tumor vascularity in pancreatic tumor patients

Claudia Alvarez Florez[1], Ana Carolina Ferreira Cardoso[1,8], Nathan Parker[2,3], An Ngo-Huang[4], Maria Q. Petzel[2], Michael P. Kim[2], David Fogelman[5], Salvador Gabriel Romero[1], Huamin Wang[6], Minjeong Park[7], Matthew H.G. Katz[2], and Keri L. Schadler[1]

Accompanying protocol 2014-0702

# Preoperative rehabilitation during neoadjuvant therapy for pancreatic cancer: a pilot study

**Study Chair:** Matthew Katz, M.D.

**Co-Chairs**: An Ngo-Huang, D.O.; Holly Holmes, M.D.; David Fogelman, M.D.

**Collaborators**: Jason Fleming, M.D.; Jeffrey E. Lee, M.D.; Gauri Varadhachary, M.D., MBBS; Rachna Shroff, M.D.; Robert Wolff, M.D.; Sunil Sahai, M.D.; Michael Overman, M.D.; Xuemei Wang, M.S., Daniel P. O'Connor, PhD (University of Houston)

## I. BACKGROUND

Pancreatic cancer is a disease that commonly afflicts older Americans and is often associated with frailty, a syndrome characterized by progressive loss of function along with the development of disability, malnutrition, and possibly impaired cognition. Frailty can be clinically identified according to clinical criteria including self-reported physical exhaustion, weak grip strength, weight loss, slow walking speed, and low physical activity [1]. Like age and comorbidity, frailty has been associated with adverse outcomes following pancreatic surgery [2].

By addressing the clinical manifestations of frailty, rehabilitation measures have been used in an attempt to improve outcomes of patients with pancreatic cancer who undergo potentially curative surgical resection. Rehabilitation regimens have typically been instituted following surgery. Home-based, postoperative rehabilitation programs as simple as structured walking regimens have been found to improve post-surgical outcomes [3].

Although postoperative rehabilitation programs may be successful, a growing body of scientific evidence supports the optimization of health, performance and nutrition of cancer patients *prior to* the initiation of anticancer therapies. ***Cancer prehabilitation*** represents a process on the continuum of care that occurs between the time of cancer diagnosis and the beginning of acute treatment, includes physical and psychological assessments that establish a baseline functional level, identifies impairments, and provides targeted interventions that improve a patient’s health to reduce the incidence and the severity of current and future impairments [4]. Prehabilitation interventions have been used to decrease morbidity, improve physical and psychological health outcomes, increase the number of potential treatment options, decrease hospital readmissions, and reduce both direct and indirect healthcare costs attributed to cancer [4].

We currently administer chemotherapy and/or chemoradiation, followed by a preoperative rest period, to most patients with potentially curable pancreatic cancer prior to intended pancreatectomy [5, 6]. One of the primary goals of this approach is to select patients with both physiology and tumor biology most appropriate for surgical resection. For our patients with potentially curable pancreatic cancer, the preoperative therapy period provides a perfect time window within which a formal prehabilitation program could be used in an attempt to improve perioperative and oncologic outcomes. Treatment tolerance and fatigue that affect exercise program adherence (and thus feasibility) may vary across chemotherapy, chemoradiation, and the preoperative rest period. To date, no formal rehabilitation program has been established or evaluated in these preoperative settings for patients with potentially resectable pancreatic cancer.

**Prior Data from MD Anderson Cancer Center**
Our group evaluates over 150 patients with resectable pancreatic cancer each year, of whom 80% receive preoperative therapy and would be considered potential candidates for this study.

We have completed a prospective study of the incidence of frailty among patients with pancreatic cancer at our institution. As part of this study, 26% of patients with pancreatic cancer of any stage met Fried Criteria for frailty [1]. Furthermore, the study, which

required completion of both the frailty assessment and a number of different survey tools (including those included in the present study) accrued rapidly with no real barriers to accrual identified in this patient population.

## II. OBJECTIVES

**Primary Objective:**
To establish the feasibility of instituting a home-based, provider-supervised "prehabilitation" program during neoadjuvant chemotherapy and chemoradiation and preoperative recovery among patients with potentially curative pancreatic cancer at a quaternary care institution.

**Secondary Objectives**:

- To assess the feasibility and compliance of nutrition intervention among these patients.
- To assess patients' tolerance for specific exercises and ability to advance the exercises.
- To assess patients' overall ability to complete study materials.

## III. STUDY ENDPOINTS

1) **Primary Endpoint:** Feasibility, based on adherence to the exercise intervention
2) **Secondary Endpoints:** Adherence to the nutrition intervention and completion of study materials.

## IV. INCLUSION CRITERIA

1) Pancreatic adenocarcinoma, biopsy-proven or suspected
2) Scheduled for intended pancreatectomy, > = 6 weeks until planned resection
3) Scheduled for neoadjuvant chemotherapy and/or chemoradiation for pancreatic cancer
4) Able to understand the description of the study and willing to participate.
5) Able to understand the exercise intervention and able to maintain a daily exercise log.
6) Participant must have telephone access and agree to engage with telephone access with the research personnel.

## V. EXCLUSION CRITERIA

1) Non-English speaking
2) Unable to complete the baseline assessment questionnaires or functional assessments
3) Underlying unstable cardiac or pulmonary disease or symptomatic cardiac disease (New York Heart Association functional class III or IV).
4) Recent fracture or acute musculoskeletal injury that precludes the ability to weight bear fully on all 4 limbs in order to participate in an exercise intervention.
5) Numeric pain rating scale of ≥7 out of 10
6) Myopathic or rheumatologic disease that impacts physical function

## VI. STUDY PLAN

### *Schema*

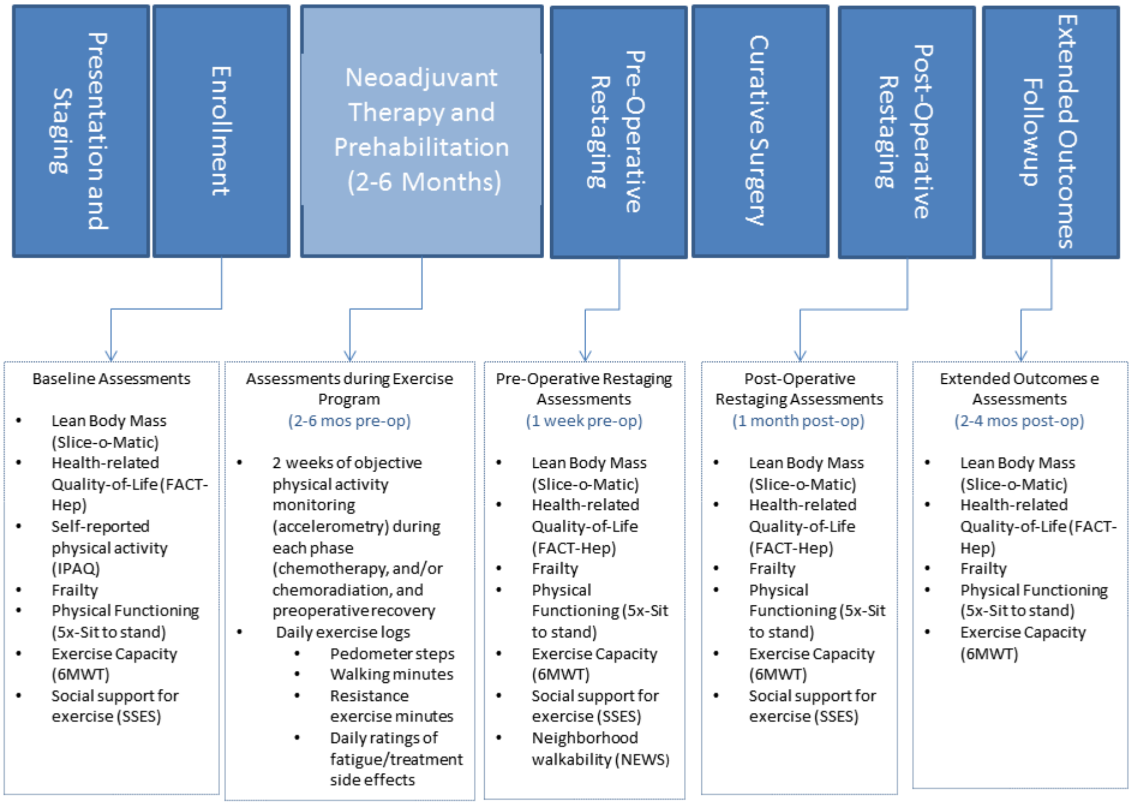


### *Pre-Enrollment Screening*

Patients will have a history and physical exam performed by the enrolling physicians to determine potential eligibility for the protocol. All patients in the surgical oncology clinic who are believed to be eligible will then be screened for enrollment to this trial using the *Physical Activity Readiness Questionnaire (PAR-Q)* and the first question of the Patient Reported Outcomes Measurement Information System *(PROMIS) Physical Function 12a Short Form*.

PAR-Q Screening

- Patients who affirmatively answer questions 2, 3, 4 on the PAR-Q will be ineligible for enrollment.
- Patients who affirmatively answer questions 1 or 6 on the PAR-Q will require clearance by an internal medicine physician (co-PI or other) prior to enrollment.  Most patients who are surgical candidates will be referred to the Internal Medicine Perioperative Assessment Center (IMPAC) Clinic close to

their pre-enrollment screening visit, as a part of routine, standard care. If they have been seen by IMPAC already, the medical co-PI will evaluate the IMPAC note and make a decision as to appropriateness for enrollment. If they have not been seen by IMPAC yet, the medical co-PI will evaluate the patient or facilitate any already scheduled IMPAC evaluation and make a final decision thereafter.

- Patients who affirmatively answer question 5 on the PAR-Q will require clearance by a PM&R physician (co-PI or other) prior to enrollment.
- Patients who affirmatively answer question 7 will require clearance by a co-PI prior to enrollment.

PROMIS Physical Function 12a Short Form Screening

- Patients who negatively answer the PF_Screener question will be ineligible for enrollment.

***Final Enrollment***

All patients will be consented prior to conducting any study related procedure and will be enrolled after they have met all screening and inclusion/exclusion criteria. At that time, the *FACT-Hep* quality of life instrument and baseline frailty assessment will be administered.

Frailty will be assessed using validated criteria, and defined as an abnormality in 3 or more of 5 markers [1]: 1) low physical activity, by self-report using *the International Physical Activity Questionnaire short form*, 2) exhaustion, by self-report using a score of 2 or 3 on either of two exhaustion items from the Center for Epidemiologic Studies Depression Scale (CES-D), 3) weight loss, defined as a loss of 3 kilograms in the prior 3 months, 4) weak grip strength, using a hand-held dynamometer and based on age- and gender-based normative values, and 5) slow gait speed, based on a cutoff of 3.2 seconds or more to complete a 3-meter walk.

***Initial Assessment of Exercise Tolerance***

All enrolled patients will complete the remainder of the PROMIS Physical Function 12a Short Form and participate in two physical screening tests to determine participation in the exercise and nutrition interventions and to evaluate exercise tolerance.

- PROMIS questions PF6, PFC29, PFA55, PFC53, PFA9 and PF_23 must be answered with a 5, 4 or 3 to participate in the exercise and nutrition interventions.
- Any answer of 1 or 2 on the PROMIS will result in the patient being excluded from participation.
- Patients must be able to perform the following tests to participate in the exercise and nutrition intervention. Patients who cannot perform both tests will be excluded from participation.
    - 5x sit-to-stand
    - 6 minute walk test (6MWT)

***Exercise Intervention***

The American College of Sports Medicine exercise guidelines for cancer survivors encourage a combination of 150 minutes of moderate-intensity aerobic activity and two

to three weekly sessions of strength training but caution that exercise programs should be adapted to the needs of each cancer survivor [7]. These exercises may be completed as part of a home-based rehabilitation and exercise program. For example, a home-based exercise program incorporating a walking and gentle strengthening program was tolerable and had positive effects on fatigue and physical function in stage IV lung and colorectal cancer patients [8]. The Rapid, Easy, Strength Training exercise protocol used in this study at the Mayo Clinic targeted multiple major muscles groups in the upper and lower limbs [8].

The home-based exercise program in this current study will incorporate both aerobic and strengthening program components at a less rigorous level than the exercise guidelines recommended by the American College of Sports Medicine, in order to ensure safety and encourage adherence. ***The exercise regimen will include A) a graded resistance/strengthening program and B) a walking program conducted during the preoperative treatment period.***

- An MDACC physical medicine and rehabilitation (PM&R) physician will supervise the conduct of the exercise intervention.

- A Kinesiology PhD candidate with an American College of Sports Medicine Personal Trainer certification and a research staff member will implement the exercise intervention, including demonstration of strengthening exercises upon verification of participation.

- All exercises will be clearly described in an exercise demonstration video (produced specifically for this program) and within the study booklet provided to all patients (attached). In addition, the exercises will be described and demonstrated in-person by the study staff. The demonstration video, photographs and descriptions of exercises in the study booklet, and in-person demonstrations will all include proper instructions for setting up resistance tubes and safety tips for each exercise. The study booklet will also include exercise logs in which the patients should record their daily exercise activity. Participants will choose to record daily exercise using these paper logs or using a secure MD Anderson electronic system.

- The preoperative period varies in terms of both the types of treatments that patients undergo and the length of therapy. Approximately 70% of patients with technically resectable tumors undergo chemotherapy (6-12 weeks) followed by chemoradiation (2-6 weeks) and then a recovery period (4-6 weeks) before surgery. Approximately 20% of patients undergo chemoradiation (2-6 weeks) and then a recovery period (4-6 weeks) before surgery. Approximately 10% of patients undergo chemotherapy (6-12 weeks) and then a recovery period (4-6 weeks) before surgery. The exercise intervention will be performed throughout the treatment course.

**A. Resistance/strengthening**

**All participants will be instructed to perform resistance/strengthening exercises for a 30-minute period at least twice a week**. The goal is to achieve moderate

exercise intensity (12-13 on the Borg Rating of Perceived Exertion (RPE) scale, included in patients' study booklets).The strengthening exercises used in this study are designed to engage proximal upper body muscles, shoulder muscles, abdominal muscles, back extensor muscles, and hip and leg muscles. Strengthening these muscle groups will develop the participant's trunk control and balance, which we believe will help with mobility during the prehabilitation phase and in the post-operative period. Furthermore, maintaining and developing the shoulder, back and leg muscles are important for transitional activities such as transferring into and out of bed, which is important after an extensive abdominal surgery such as a pancreatectomy.

**Participants will be instructed to perform any 8 strengthening exercises for a total duration of 30 minutes.** Each patient will be issued a set of 3 resistance exercise tubes with which the exercises can be performed. The strengthening exercises consist of seated and standing weight-bearing exercises. A variety of exercises targeting different muscle groups will be prescribed to minimize plateauing, and to generate increased interest and motivation (and thus, adherence). Participants will perform 3 sets of 8-12 repetitions each of each exercise. Once they are able to perform 3 sets of 12 repetitions of an exercise, they will graduate to the next level of increasing resistance as denoted by the color of the resistance tube used.

Prior to initiating the strengthening exercises, each participant will initiate a series of 5 *standing warm-up exercises* before each resistance exercise session that will help with balance.

Participants will select the 8 exercises to be performed from 19 strengthening exercises that include:

- *Upper body strengthening exercises* consist of seated and standing exercises to strengthen the deltoids, triceps, biceps, and rotator cuff. These exercises will include (but are not limited to) bench press, chest press, and biceps curls using resistance tubes.

- A*bdominal strengthening exercises* that will be performed while seated or standing using resistance tubes, which will be more tolerable as older adults may not be able to tolerate the supine-position abdominal exercises.

- *Lower body strengthening exercises* that consist of s*eated and standing* exercises to strengthen the gluteus musculature, quadriceps, hamstrings, and gastrocnemius-soleus complex. These exercises will include (but are not limited to) leg lifts, leg extensions, hip extensions, and hip abductions. Most exercises will be performed using resistance tubes.

Participants will record the date, resistance (color of resistance tube used), number of repetitions in each set, and their perceived intensity of a resistance exercise session based on the Borg Rating of Perceived Exertion (RPE) scale (the scale will be included in the participants' exercise program packets). They will also record the total time spent on their strengthening program after each exercise session. When each patient completes the exercise portion of the study, they will be asked which exercises they preferred.

The participants will be *encouraged* to complete an additional 5 minutes of stretching before and after resistance exercise sessions. Instructions and photographs of stretches targeting muscles in the upper body, back, and legs are included in the exercise program packets.

**B. Walking program**

**The walking program will consist of walking briskly for a duration of 20 - 30 minutes per day, with a frequency of at least 3 times per week.** The goal is to achieve a moderate intensity of exercise (Borg RPE of 12 - 13). If the participant is unable to tolerate walking briskly, they will be instructed to walk at a tolerable pace for 20 to 30 minutes during each walking session. Each participant will be provided a pedometer and will be instructed to record the number of steps taken each day in addition to the total time spent during each walking session.

The participants will be provided with exercise logs (both electronic, using MD Anderson's Research Electronic Data Capture - REDCap – system, and paper forms) in which they will record their daily physical activity. Participants will choose whether to record daily physical activity and exercise activities using REDCap or paper logs.

***Emergency contacts***

The booklet provided to all patients will include emergency 24/7 contacts (physician, co-PI) in the event of an acute musculoskeletal injury or cardiopulmonary distress. In the event that this occurs, the patient will be managed with an appropriate medical referral. If the patient develops a musculoskeletal injury, they will be triaged to speak with one of the PM&R research team members. Patients who develop disease progression and/or have significant cancer treatment-associated toxicity will also be counseled on the continuation of this exercise program accordingly.

***Adherence/compliance***

To monitor for adherence and screen for any exercise intolerance or exercise barriers, all participants will be contacted by the research staff via telephone every two weeks. The research staff will be provided with a list of screening questions (attached) to address exercise adherence and assess for the need to advance the strengthening program. The research staff will also implement behavior interventions to encourage continued adherence to the exercise program that will include education on the benefits of exercise and physical activity.

It is anticipated that a fully adherent participant would be able to complete at least 60 minutes of moderate-intensity walking and 60 minutes of moderate-intensity resistance exercise per week. Adherence to the program will be based on completion of a variety of strengthening exercises (eight or more per session) and the total time spent performing both the walking and strengthening components. A participant who is able to complete 60 minutes of walking and 60 minutes of resistance exercise per week during each phase of neoadjuvant therapy will be classified as 100% adherent during that phase.

***Distress Plan***

In the event that a patient has difficulty with any study-related questionnaires or where the patient requests assistance for distress or where a practitioner determines that there is cause for concern, patients will be referred for assessment to a certified professional for assistance in managing their distress.

***Equipment provided***

- Notebook for the exercise activity logs and written/photo exercise guides
- Pedometer: $20 Omron HJ-321 TriAxis pedometer: http://www.amazon.com/Omron-HJ-321-Tri-Axis-Pedometer-Black/dp/B007ZWIJR2
- Resistance exercise tube set (set of 5, patients will be provided with the 3 with lowest resistance) including door anchor, ankle strap, and handles, $32.99 per set)

***Nutrition intervention***

Within 7 days of enrollment, a registered dietitian (RD, ie the study dietitian or delegate RD) will conduct a complete nutritional assessment. Each subject will be then be provided with individualized recommendations for calorie, protein, and fluid intake, tools for tracking intake (ie, diet log), as well as goals for weight maintenance, weight gain, or weight loss depending on stage of treatment/recovery. Patients will also be instructed to eat a high protein snack/meal/shake (~20 g) within one hour after strengthening exercises.

As is current “standard of care” for our patients who undergo pancreatic surgery, all patients will also receive instruction on the use of an immune enhancing oral supplement (Impact Advanced Recovery) for 5 days preoperatively (preop days 6 through 1).

When the research staff contacts the participants via telephone to monitor their exercise program, they will also be asked questions regarding their compliance with the nutritional plan. The research staff will be provided with a list of questions to assess patient understanding of and ability to comply with nutrition recommendations. If patients are non-compliant or have difficulty comprehending the nutritional program, the RD will be notified and RD will follow up with the patient for reeducation or adjustment of goals. To monitor compliance with immune enhancing oral supplement, the patient/family will be interviewed following surgery to determine consumption.

## VII. Statistics

This is a pilot study to assess the feasibility of a provider-supervised prehabilitation program among pancreatic patients who are undergoing neoadjuvant therapy. The prehabilitation program will include both exercise and nutrition interventions, where the exercise intervention will incorporate resistance/strengthening and walking programs. Adherence will be defined as the ability of each patient to complete ≥60% of the planned exercise intervention based on the total time spent performing the exercises relative to the total duration of therapy or total duration of recovery for a given phase. The primary endpoint, feasibility, will be established if ≥ 60% of the patients are adherent during a phase of therapy. That is, the prehabilitation program is deemed as feasible during a specific phase of therapy if 60% or more of the participants complete at least 60% of the weekly prescriptions for 60 minutes of walking and 60 minutes of strengthening

exercises during that phase of therapy. The calculation will account for the potential difference in the length of therapy among patients. The target weekly total exercise hour will be 120 minutes (including 60 minutes of walking and 60 minutes of strengthening exercises), thus we can derive the total prescribed hour for a given patient in a given phase considering his/her total treatment or recovery duration for that phase. Then, based on the recorded total hours of exercise intervention for that patient, we can calculate the % of completion for that patient in a given phase. If the % is at least 60% for a phase, we would define that patient as “adherent” for that phase. For the primary analysis, we will analyze the % adherence by phase, where “phase” could be chemotherapy phase, chemoradiation phase or recovery phase. As a secondary analysis, we will further differentiate whether patients receive chemoradiation from start or chemoradiation is given following chemotherapy and analyze these two subgroups of patients separately. Similarly, we will differentiate patients in recovery phase based on the therapies they receive prior to the recovery (i.e., chemotherapy only, chemoradiation only or chemotherapy plus chemoradiation).

The study was originally designed to enroll 50 patients and as of August 2015, 20 patients have been enrolled under this initial design. We have placed the study on hold until these attached revisions are made because we have encountered several issues with the first design and have identified solutions to these problems. The primary issue is that, under the old design, we would assess the exercise intervention in a 6-week period regardless of the treatment phase. Initial analysis of the data has shown us that patients’ adherence depends to a large degree on the phase of therapy. For example, patients seem to be less compliant with exercise during chemotherapy than they are during either chemoradiation or rest. Future trials will require us to time the intervention precisely and optimize the intervention during preoperative therapy, so we need to know the best “phase” (or phases) of therapy to deliver the exercise intervention. This trial will allow us to gain a more accurate and better understanding about patients’ adherence during each phase so as to guide the focus for future studies.

We have also changed a couple of the survey tools and assessments we will use in this new design, as described below. We added tools to help us explore barriers to exercise and we improved a physical assessment from one with low resolution to one with higher resolution (and, it is used more frequently in the literature).

Under the revised design, we will enroll 50 new patients and evaluate the feasibility of the exercise intervention through assessing their adherence rate during each phase of the neoadjuvant therapy. We assume that among the 50 patients, 35 patients will receive chemo followed by chemoradiation, recovery and then surgery; 10 patients will receive chemoradiation followed by recovery phase and then surgery; and 5 patients will receive chemotherapy followed by recovery phase and then surgery. Therefore, overall we will have 40 patients’ data for assessing the adherent rate in the chemotherapy phase, 45 patients’ data for assessing the adherent rate in the chemotheradiation phase and 50 patients’ data for the recovery phase. Thus, with a sample size of 40, 45 and 50 and assuming an adherence rate of 60%, the exact 95% confidence interval will be (43%, 75%), (44%, 74%) and (45%, 74%), respectively.

Descriptive statistics, such as frequency (percentage) and median (range) will be used to summarize the data, including patient demographics, clinical features, comorbidity,

symptoms burden and quality of life. We will also summarize patients' tolerance for specific exercises, their ability to advance the exercises and the motivating/prohibiting factors for them to complete the study materials. The rate of adherence will be estimated for each phase, along with the exact 95% confidence interval. Similarly, the proportion of patients with frailty (i.e., abnormality in 3 or more of 5 markers), perioperative complications (yes/no; high/low grade) and physical/functional measures (yes/no for completing the 5x sit-to-stand test and the 6-minute walk test) will be estimated, along with the exact 95% confidence intervals. As an exploratory analysis, we will fit a multiple logistic regression model to assess the association between patient characteristics and socioecological characteristics (social support for exercise and neighborhood walkability) and adherence (yes/no) for each phase. In addition, paired t-test or Wilcoxon signed rank test will be used to assess the change of Fact-Hep, Frailty, physical functioning, and exercise capacity measures from baseline. We will also fit a linear mixed model to assess the change of each measurement over time (i.e., at enrollment, pre-op and post-op visit), while accounting for the correlation among multiple observations from the same patient.

***Study Measures***

Frailty will be measured based using validated criteria, and defined as an abnormality in 3 or more of 5 markers [1]: 1) low physical activity, by self-report using the International Physical Activity Questionnaire short form, 2) exhaustion, by self-report using a score of 2 or 3 on either of two exhaustion items from the Center for Epidemiologic Studies Depression Scale (CES-D), 3) weight loss, defined as a loss of 3 kilograms in the prior 3 months, 4) weak grip strength, using a hand-held dynamometer and based on age- and gender-based normative values, and 5) slow gait speed, based on a cutoff of 3.2 seconds or more to complete a 3-meter walk.

Perioperative complications will be prospectively graded and recorded using the modified Accordion scale [9, 10].

**Pancreatic ductal adenocarcinoma** (PDAC) Clinical Features: Additional clinical disease characteristics will be determined based on laboratory studies (albumin, prealbumin, c-reactive protein, creatinine, bilirubin, serum CA 19-9).

Demographic factors will include age, sex, race/ethnicity, place of residence, and presence of a primary caregiver.

Comorbidity will be determined prospectively using the Adult Comorbidity Evaluation-27 (ACE-27), a validated grading system that is predictive of prognosis in other cancer populations.

Symptom burden and quality of life (QOL) will be assessed using the Functional Assessment of Cancer Therapy-Hepatobiliary (FACT-Hep) quality of life and symptom index.

Physical functioning measures and exercise capacity: PROMIS SF v1.0 – Physical Function and 5x sit-to-stand test and 6-minute walk test

Lean body mass as measured by Slice-o-matic version 5.0.

Treatment side effects and fatigue: Participants will complete 2 items from the FACT-Hep daily, included on exercise logs. These items include GP5 ("I am bothered by side effects of treatment) and H17 ("I feel fatigued").

Physical activity will be measured using both objective monitoring and self-report.

> Objective physical activity: Participants will undergo 2 weeks of objective monitoring using Actigraph GT3X accelerometers during each phase of neoadjuvant therapy (chemotherapy and/or chemoradiation and the preoperative recovery period) to measure total, light, and moderate-to-vigorous physical activity.
>
> Self-reported physical activity: Participants will report physical activity using the IPAQ short form (administered as part of the frailty assessment) and in daily exercise logs.

Social support for exercise will be measured using the Social Support for Exercise Scale.

Neighborhood walkability for exercise will be measured using subscales E, F, G, and H from the Neighborhood Environment Walkability Survey – Abbreviated (NEWS-A). NEWS-A subscales will be administered to measure walkability for exercise in each neighborhood/area in which a patient lives during preoperative therapy.

Qualitative interviews will be conducted with a subsample of patients who have completed the exercise program. The purpose of these interviews is to examine program satisfaction and influences and outcomes related to adherence during the preoperative exercise program. Specifically, we seek to understand factors that may have motivated patients to exercise, differences in and barriers to adherence during different treatment phases, perceived physical and emotional benefits from exercise, program satisfaction, and suggestions for program improvement.

All patients who have completed the preoperative exercise program will be considered for enrollment on this protocol. Following pre-operative restaging appointments (for patients who do not undergo surgical resection) or discharge from MD Anderson after surgical resection (approximately 3-4 weeks after surgical resection) patients who are deemed interview candidates will be called by phone (by Nathan Parker, a PhD candidate in kinesiology and Graduate Research Assistant in the Department of Surgical Oncology) to explain the purpose of interviews and steps that will be taken to analyze data and protect its confidentiality. During these calls, patients will be questioned regarding their interest in this interview, either at the time of the initial phone call or at a future time to be scheduled. Structured interviews will last approximately 30 minutes and will be audio-recorded using Microsoft Lync software. Audio files will be transcribed verbatim in Microsoft Word. De-identified, transcribed interviews will be managed using QSR Nvivo 10 software and analyzed for emergent themes by Parker and an additional PhD Kinesiology student with experience in qualitative data analysis. Interviews will be coded for emergent themes (first independently by each coder, then convergently as a coding pair) using a constant comparison approach. This approach allows for the

development of explanations through patterns but also allows coders to utilize theory and prior knowledge or research to answer research questions [11].

We aim to recruit roughly equal numbers of male and female participants and participants who met/exceeded or did not meet baseline recommendations for walking and resistance exercise. However, we recognize that differences in interest and availability can affect recruitment, and we may have imbalances in these proportions. Recent qualitative research studies examining influences and outcomes related to physical activity among cancer survivors have reached saturation of themes after interviewing five [12] and nine [13] participants. Ten of the 20 patients enrolled in the original 2014-0702 prehabilitation protocol underwent surgery, and we expect approximately 50% of the 50 patients who have enrolled or will enroll in the current (revised) protocol to undergo surgery. Therefore, we will have access to approximately 35 patients who are possible candidates for this study. We expect to conduct approximately 12-15 interviews among this group. Qualitative analysis of interview transcripts will occur on a rolling bases, however, and we will end recruitment and cease interviewing upon reaching saturation of qualitative themes.

The following is a list of interview topics and questions:

**A. *Past exercise experience***

- Please describe your experience with aerobic exercise (like walking) prior to starting on our exercise program.
- Please describe your experience with strengthening exercise (like weight training) prior to starting on our exercise program.
- How did your past exercise experience affect your participation in this exercise program?

**B. *Motivation to exercise***

- What motivated you to exercise during this program?
  - Potential probing questions:
    - What roles did diagnosis or goals for treatment play in motivating you to exercise during this program?
    - What roles did your family or friends play in motivating you to exercise during this program?
- Did your motivation to exercise change over the course of this program?
- How would you describe your motivation to exercise now that the program is finished and you've had surgery?

**C. *Perceived exercise on the program***

- Did you feel like you were able to exercise enough during this program?
- How did your ability to exercise change from [modify as appropriate] chemo to chemotherapy and to preoperative rest?

**D. *Barriers to exercise***

- Please describe any factors that made it difficult for you to exercise during this program.
  - Potential probing questions:

            - Describe the role that scheduling or travel related to preoperative treatments played in your ability to exercise during this program.
            - What aspects of your support system or resources at home made it difficult for you to exercise?
            - What additional types of support or resources helped you exercise or exercise more?
- Did these factors change at all from [modify as appropriate] chemo to chemotherapy and to preoperative rest?

### *E. Facilitators of exercise*

- Please describe any factors that made it easy for you to exercise during this program.
    - Potential probing questions:
        - What aspects of the program made it easy for you to exercise?
        - What aspects of your support system or resources at home made it easy for you to exercise?
- Did these factors change at all from [modify as appropriate] chemo to chemotherapy and to preoperative rest?

### *F. Perceived outcomes*

- How do you think participating in this program and exercising during your preoperative therapy may have benefitted you?
    - Potential probing questions:
        - How did participating in this program affect the support you felt you had during your preoperative treatments?
        - How did participating in this program affect you physically?
        - How did participating in this program affect you emotionally?
        - How did participating in this program affect your ability to tolerate therapy?
        - How has the exercise program affected your recovery following surgery? [OR] How do you expect the exercise program to affect your recovery following surgery?

### *G. Satisfaction and suggestions for program improvement*

- How would you describe your overall satisfaction with this exercise program?
- What aspects of the program did you particularly appreciate or enjoy?
- What aspects of the program would you change?
    - Potential probing questions:
        - How would these changes have affected your ability to exercise or your enjoyment of the program?

## VIII. Data Security and Confidentiality

Study will be monitored by the Principal Investigator (PI). Study personnel will record study data on protocol-specific forms and/or electronic forms to be completed and maintained at MD Anderson. De-identified data for exercise/physical activity and related influence and outcome measures will provided to and used for analyses in collaboration with Daniel P. O'Connor, PhD, Associate Professor of Kinesiology at the University of Houston Department of Health and Human Performance. Dr. O'Connor advises Nathan Parker, a PhD student in Kinesiology and Graduate Research Assistant for Dr. Katz in the Department of Surgical Oncology, who will be using data from this study for his doctoral dissertation project. All research activities involving these data will be conducted in direct collaboration with the study PI. Any study-related documents with identifiable patient information will be maintained at MD Anderson in an appropriately secure manner per institutional policy.

Data will be retained after termination of this study. Data will be stored on password protected, encrypted hard drives inside of an institutional firewall. Records will be destroyed when the final manuscript is published. Audio recordings from qualitative interviews will not contain any identifiable information and will be stored temporarily with password protection on an encrypted volume behind a firewall. Only the PI, collaborators, and research staff will have access to the recordings. Audio recordings will be destroyed immediately after transcription. De-identified transcriptions will be stored in the same location and destroyed when the final manuscript is published. Data will be stored for up to 2 years after termination of this study, at which point all documents will be destroyed by a means that assures that all files are irretrievable.

Study records will be stored in a secured storage area, and electronic data will be secured with password protection on an encrypted volume behind a firewall. Only the PI, collaborators, and research staff will have access to the data.

## IX. References

1. Fried, L.P., et al., *Frailty in older adults: evidence for a phenotype.* J Gerontol A Biol Sci Med Sci, 2001. **56**(3): p. M146-56.
2. Dale, W., et al., *Geriatric assessment improves prediction of surgical outcomes in older adults undergoing pancreaticoduodenectomy: a prospective cohort study.* Ann Surg, 2014. **259**(5): p. 960-5.
3. Yeo, T.P., et al., *A progressive postresection walking program significantly improves fatigue and health-related quality of life in pancreas and periampullary cancer patients.* J Am Coll Surg, 2012. **214**(4): p. 463-75; discussion 475-7.

4. Silver, J.K. and J. Baima, *Cancer prehabilitation: an opportunity to decrease treatment-related morbidity, increase cancer treatment options, and improve physical and psychological health outcomes.* Am J Phys Med Rehabil, 2013. **92**(8): p. 715-27.
5. Cooper, A.B., et al., *Role of Neoadjuvant Therapy in the Multimodality Treatment of Older Patients with Pancreatic Cancer.* J Am Coll Surg, 2014.
6. Tzeng, C.W., et al., *Defined clinical classifications are associated with outcome of patients with anatomically resectable pancreatic adenocarcinoma treated with neoadjuvant therapy.* Ann Surg Oncol, 2012. **19**(6): p. 2045-53.
7. Schmitz, K.H., et al., *American College of Sports Medicine roundtable on exercise guidelines for cancer survivors.* Med Sci Sports Exerc, 2010. **42**(7): p. 1409-26.
8. Cheville, A.L., et al., *A home-based exercise program to improve function, fatigue, and sleep quality in patients with Stage IV lung and colorectal cancer: a randomized controlled trial.* J Pain Symptom Manage, 2013. **45**(5): p. 811-21.
9. Porembka, M.R., et al., *Quantitative weighting of postoperative complications based on the accordion severity grading system: demonstration of potential impact using the american college of surgeons national surgical quality improvement program.* J Am Coll Surg, 2010. **210**(3): p. 286-98.
10. Strasberg, S.M., D.C. Linehan, and W.G. Hawkins, *The accordion severity grading system of surgical complications.* Ann Surg, 2009. **250**(2): p. 177-86.
11. Bernard, H.R. and G.W. Ryan, *Analyzing Qualitative Data: Systematic Approaches.* . 2010, Newbury Park, CA: Sage Publications.
12. Mas, S., X. Quantin, and G. Ninot, *Barriers to, and Facilitators of Physical Activity in Patients Receiving Chemotherapy for Lung Cancer: An exploratory study.* J Palliat Care, 2015. **31**(2): p. 89-96.
13. Brunet, J., et al., *A qualitative exploration of barriers and motivators to physical activity participation in women treated for breast cancer.* Disabil Rehabil, 2013. **35**(24): p. 2038-45.
